# Efficient Distributed Method for NLOS Cooperative Localization in WSNs

**DOI:** 10.3390/s19051173

**Published:** 2019-03-07

**Authors:** Shiwa Chen, Jianyun Zhang, Yunxiang Mao, Chengcheng Xu, Yu Gu

**Affiliations:** 1 College of Electronic Engineering, National University of Defense Technology, Hefei 230037, China; zjy921@sina.com (J.Z.); myxeei316@sina.com (Y.M.); xuchengcheng17@nudt.edu.cn (C.X.); 2 State Key Laboratory of Pulsed Power Laser Technology, National University of Defense Technology, Hefei 230037, China; terryguyu@163.com

**Keywords:** wireless sensor networks (WSN), cooperative localization, non-line-of-sight (NLOS), alternating direction method of multipliers (ADMM), convex relaxation

## Abstract

The accuracy of cooperative localization can be severely degraded in non-line-of-sight (NLOS) environments. Although most existing approaches modify models to alleviate NLOS impact, computational speed does not satisfy practical applications. In this paper, we propose a distributed cooperative localization method for wireless sensor networks (WSNs) in NLOS environments. The convex model in the proposed method is based on projection relaxation. This model was designed for situations where prior information on NLOS connections is unavailable. We developed an efficient decomposed formulation for the convex counterpart, and designed a parallel distributed algorithm based on the alternating direction method of multipliers (ADMM), which significantly improves computational speed. To accelerate the convergence rate of local updates, we approached the subproblems via the proximal algorithm and analyzed its computational complexity. Numerical simulation results demonstrate that our approach is superior in processing speed and accuracy to other methods in NLOS scenarios.

## 1. Introduction

Wireless-sensor-network (WSN) technology has rapidly developed because of its convenience and prospects. It can significantly improve living quality in many important fields, such as environment monitoring [1] and surveillance [2], vehicle tracking [3,4], exploration [5,6], and other sensing tasks [7]. According to Reference [1], WSNs extend the human ability “to monitor and control physical world”. This means that WSNs provide a more intelligent way to link the physical world with humans. It is worth noting that the positions of sensor nodes are key information for WSNs to fulfil various tasks. Therefore, as a preliminary task, cooperative localization has aroused increasing interest, especially in indoor scenarios where satellite communications cannot be employed. In general, cooperative localization has two main categories: rangefree methods and range-based methods [8]. Rangefree methods are easier to implement, but their accuracy is lower than range-based methods [9]. The most common range-measurement techniques of range-based localization are based on received signal-strength indicator (RSSI) [10] and time of flight (TOF) [11]. The RSSI technique has a lower cost than the TOF technique, but the latter has higher accuracy. In this paper, we mainly study the localization problem based on range-based technology. Some related work in this field is listed as follows.

### 1.1. Related Work

The maximum-likelihood (ML) problem of sensor cooperative localization is a complicated nonconvex problem of high dimensionality. It is quite difficult to obtain an optimal solution. Most existing approaches try to find a feasible solution by applying relaxation methods to the original nonconvex problem. Among them, the most typical ones are listed. Semidefinite programming (SDP) relaxation for cooperative localization is proposed in References [12,13,14]. In Reference [13], the authors formulated the cooperative localization problem via graph realization theory, and derived the upper and lower bounds of the SDP objective function. In Reference [14], the authors developed three relaxations: node-based SDP (NSDP), edge-based SDP (ESDP), and sub-SDP (SSDP). NSDP and ESDP are weaker than the original SDP relaxation of Reference [12], but they both remain efficient and accurate. SSDP paves a faster way to efficiently solve a general SDP problem without sacrificing solution quality. In Reference [15], the authors proposed a novel model by combining angle information with range measurements, and transformed the model into an SDP problem. This method has excellent performance, but it also has considerably high computational complexity. The work in References [16,17] proposes second-order cone programming (SOCP) by relaxing distance constraints. The work in Reference [16] further applies SOCP to alleviate the computational burden of the standard SDP problem at the price of performance degradation. The authors in Reference [17] designed a method suited for both Gaussian and Laplacian noise environments. In addition to the aforementioned convex relaxations, some other methods also contribute to improving localization performance. In Reference [18], the authors formulated the problem as a regression problem over adaptive bases. They utilized the eigenvector of a distance affinity matrix as the initial point and implemented iterations by conjugate gradient descent. In Reference [19], the authors derived a majorization–minimization (MM) algorithm with quadratic objective function. However, all the methods or algorithms above are implemented in a centralized framework.

The centralized framework is a classical paradigm that transmits data to the central or fusion node to fulfil entire network tasks. A centralized framework is easy to implement, but the computational burden becomes extremely heavy for large-scale sensor networks. Centralized algorithms are prone to data-traffic bottlenecks among sensors around the central node [20]. As the number of sensor nodes grows, the problem size and the computational complexity in the centralized framework dramatically increase. Scale limits and time delay brought by the centralized paradigm may affect the engineering implementation of WSNs. Due to the advent of large-scale networks, it is urgent to find an effective framework to satisfy the requirements on both processing speed and localization accuracy. Therefore, the distributed paradigm has become a new tendency in this field.

Compared with the centralized paradigm, the distributed paradigm is an algorithm with all nodes performing the same type of computation. A number of distributed approaches have been proposed over the years, such as the distributed gradient descent method [21], multidimensional scaling method [22], and the sequential greedy optimization (SGO) algorithm [23]. In Reference [24], the authors developed a parallel distributed algorithm based on SOCP relaxation, but the convergence property of the algorithm was not established. The work in Reference [25] proposes a sequential method: the estimated neighbors are regarded as new anchors, which are then used to estimate other sensors. This process only stops when the whole network is covered. In Reference [23], the authors applied the SGO algorithm to ESDP and SOCP relaxation formulations. The work in Reference [26] developed an ESDP relaxation method and designed a distributed algorithm relying on the alternating direction method of multipliers (ADMM), of which the convergence property was guaranteed and proven in Reference [27]. Then, in Reference [28], the authors proposed a distributed method based on ADMM in the presence of harsh nonconvexities. In oder to guarantee that the solution converges to the optimal point, the authors established the mathematic model to choose penalty parameters. The work in Reference [20] proposed a tighter convex problem via projection relaxation and put this problem into the distributed Nesterov framework. A hybrid solution was presented in Reference [29] by combining the convex relaxations of Reference [20] and the distributed method of Reference [28]. These distributed approaches are effective and bring a controlled computational burden. Convergence performance is generally guaranteed.

However, the aforementioned distributed algorithms work only in line-of-sight (LOS) scenarios. In some practical situations, such as in forests, cities, and indoor places, most connections between sensors are non-line-of-sight (NLOS) because of the obstacles in the direct paths of signal propagation. This phenomenon can severely degrade localization accuracy if the NLOS impact is not taken into consideration. In general, two approaches are applied to alleviate NLOS propagation in localization. The first approach distinguishes LOS and NLOS connections via prior information [30,31,32]. This approach needs other measurement techniques to provide NLOS information, such as Direction of Arrival (DOA). Hence, its estimation model is less suitable for cooperative localization methods based on range-based techniques. The second approach modifies the original model by weighting and adding constraints of range-measurement errors [33,34]. This kind of approach can be applied to more general scenarios. Some representative work is listed as follows. The work in Reference [35] is “the first one to address NLOS node localization in WSN". According to three different scenarios, the authors provided three modified models of the ML estimator by adding the upper and lower bounds of range measurements. These models were solved in standard SDP problems with heuristic estimation. The work in Reference [34] proposed an ESDP method and added constraints to improve robustness. In Reference [36], the authors introduced NLOS bias parameters to the estimation model for the source-node locations and turned the model into a SDP problem. In Reference [37], the authors proposed a three-block ADMM algorithm based on the model in Reference [36]. However, this method does not decrease the size of the original problem, and the authors also mention that “it is hard to distribute the calculation" because it involves matrix projection. The work in Reference [38] proposed a distributed algorithm based on the Huber estimation of Reference [39], but it did not provide theoretical proof for the convergence property.

### 1.2. Contributions

Up to now, most NLOS mitigation techniques applied to WSN cooperative localization still utilize centralized optimum algorithms. In this paper, we propose a parallel distributed algorithm based on a tight relaxation technique to both decrease computational complexity and improve accuracy in NLOS environments.

First, we propose a modified convex model based on projection relaxation, which relaxes the original nonconvex problem into its convex envelope. This model can be applied to tough situations where NLOS connections are unidentifiable in all range measurements. According to the bounds of range measurements, we formulated the problem in the form of projection distances. Then, we relaxed this formulation into the projection on convex sets by using Cauchy–Schwartz inequality. Compared with SDP, this approach improves the decomposition property of the convex model and the accuracy of the estimation results.

Second, we developed a parallel distributed algorithm to solve the convex model. We designed a consensus form to decompose the large-scale problem into numerous local subproblems and provide the relevant theoretical proof. The proposed consensus form is more suitable for an ADMM framework. It enables each node to solve each subproblem in parallel. We derived the concrete procedure of how to handle the problem in a parallel way. The distributed algorithm had much lower computational complexity than that in existing papers about NLOS cooperative localization.

Third, we further improved the convergence rate of local updates. The local subproblems are convex and nonquadratic differentiable, which makes them less appropriate for the Newton method and interior-point algorithm. Hence, with the guarantee of Lipschitz continuity, we propose an iterative algorithm to solve the untypical convex subproblems based on the proximal method.

The paper is organized as follows. Section 2 formulates the cooperative localization problem and the corresponding convex relaxation. Section 3 presents the consensus form and solves it in a distributed way. Section 4 derives the iterative method for local subproblems. In Section 5, the simulation results are reported. Section 6 concludes the paper.

## 2. Problem Formulation

### 2.1. Mathematic Model

**Figure 1:**
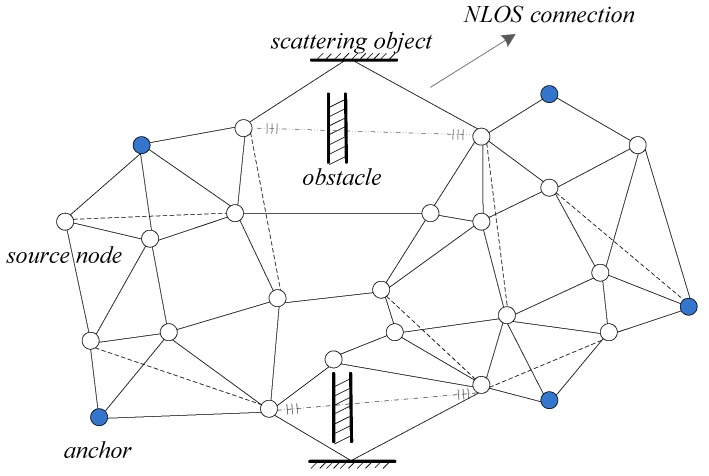
Wireless sensor network (WSN) figuration. Blue circles denote anchors; white circles denote source nodes. The physical model of non-line-of-sight (NLOS) connections is presented by *obstacle* and *scattering object*.

The mathematic model of range-based cooperative localization is described as follows. As is shown in Figure 1, consider a sensor network consisting of *N* source sensors, of which locations $x_{i}\in R^{d},\;i=1,2,\ldots ,N$ are unknown, and *M* anchor sensors of which the location $x_{k}\in R^{d},\;k=N+1,\ldots ,N+M$ is known. *d* is the coordinate dimension. Source nodes are collected in the set S={1,2,…,N}, and anchors are collected in the A={N+1,N+2,…,N+M} set. We denote the Euclidean distance between node *i* and *j* as $d_{i,j}$, and the noisy range measurement as $r_{i,j}$. In general, not every pair of nodes can communicate because the communication distance has an upper limit. We denoted this distance limit as $r_{\mu }$. The neighbor of node *i* is denoted as $j\in N_{i}$ if $r_{i,j}$ is available, i.e., $N_{i}=\left\{j|r_{i,j}\leq r_{\mu },\forall j\in S\right\},\;\forall i\in S\cup A$. We denoted the pairwise of source sensors as $\left(i,j\right)\in Z_{S}$, and the pairwise between a source sensor and an anchor as $\left(i,k\right)\in Z_{A}$. The distance between two nodes is defined as

(1) $$d_{i,j}=∥x_{i}-x_{j}∥$$

Considering the impact of NLOS propagation on distance estimation, we divided the range measurements into two sets. We used $Z_{\mathrm{LOS}}$ (and, respectively, $Z_{\mathrm{NLOS}}$) to denote the set of pairwise nodes in which connections between nodes are LOS (and, respectively, NLOS). Hence, the range measurements are defined as:

(2) $$\left\{ \begin{array}{c} r_{i,j}=d_{i,j}+n_{i,j},\left(i,j\right)\in Z_{\mathrm{LOS}}\\ r_{i,j}=d_{i,j}+\varepsilon_{i,j}+n_{i,j},\left(i,j\right)\in Z_{\mathrm{NLOS}} \end{array}\right.$$

where $n_{i,j}∼N\left(0,\sigma_{i,j}^{2}\right)$ is the measurement noise following a zero-mean Gaussian distribution with variance $\sigma_{i,j}^{2}$, and $\varepsilon_{i,j}$ is the error of the NLOS measurement that is exponentially distributed with a mean parameter $\alpha_{i,j}=\alpha_{\mathrm{NLOS}}$. The value of $\alpha_{\mathrm{NLOS}}$ depends on the NLOS propagation environment [40,41,42].

In most cases, prior information of NLOS connections, such as NLOS distribution, is unavailable. In this paper, our model was designed for such tough scenarios, i.e., NLOS connections being unidentifiable among all connections. First, since we could not distinguish which connections were NLOS, we assumed that all range measurements were NLOS. Model $r_{i,j}$ was modified as

(3) $$r_{i,j}=d_{i,j}+\varepsilon_{i,j}+n_{i,j},\left(i,j\right)\in Z_{\mathrm{NLOS}}\cup Z_{\mathrm{LOS}}$$

### 2.2. Convex Relaxation

The method in Reference [35] provided two bounds of $d_{i,j}$ in NLOS environments. The upper bound is

(4) $$u_{i,j}=r_{i,j}+2\sigma_{i,j}\geq d_{i,j}$$

the lower bound is

(5) $$l_{i,j}=r_{i,j}-4\sigma_{i,j}\leq d_{i,j}$$

For the single-constraint case, the upper and lower bounds provide an annular feasible region. Heuristic localization estimation lies on the circle with a radius of $(u_{i,j}+l_{i,j})/2$. Hence, the optimization problem of cooperative localization in an NLOS environment is modified as:

(6) $$\hat{X_{s}}=\underset{X_{s}}{\mathrm{argmin}}\sum_{\left(i,j\right)\in Z_{S}\cup Z_{A}}\;\frac{1}{2}{\left(∥x_{i}-x_{j}∥-\frac{1}{2}\left(l_{i,j}+u_{i,j}\right)\right)}^{2}$$

This model takes NLOS impact into account and uses heuristic points $(u_{i,j}+l_{i,j})/2$ to replace range measurements $r_{i,j}$. In severe environments where most connections are NLOS, range measurements may contain much false information and, thus, cannot directly be used as distance metrics. In Model (6), the bounds can neutralize most NLOS errors in range measurements. However, Problem (6) is nonconvex. In this paper, we propose a tight relaxation method rather than SDP. We used projection relaxation to obtain the convex envelope of the original nonconvex problem. The theoretical derivation is given as follows.

First, we rewrote the fundamental part of Problem (6) in the form of a squared distance of the projection on a certain set by using Cauchy–Schwartz inequality.

The proof is given as follows:

If $∥b∥=d_{0}$, we have

(7) $$\begin{array}{cc} {\left(∥a∥-d_{0}\right)}^{2} & ={∥a∥}^{2}-2∥a∥∥b∥+{∥b∥}^{2}\\ & \leq {∥a∥}^{2}-2a^{T}b+{∥b∥}^{2}\\ & ={∥a-b∥}^{2} \end{array}$$

Then, function

(8) $$f\left(a,d\right)={\left(∥a∥-d_{0}\right)}^{2}$$

can be rewritten as

(9) $$f\left(a,d\right)=\underset{∥b∥=d_{0}}{\inf}{∥a-b∥}^{2}={\mathrm{dist}}^{2}\left(a,B\right)$$

where $B=\{b|∥b∥=d_{0}\}$ is a nonconvex set. Hence, the original problem can be written in a simpler form:

(10) $$\min\;\sum_{\left(i,j\right)\in Z_{S}\cup Z_{A}}\frac{1}{2}{\mathrm{dist}}^{2}\left(x_{i}-x_{j},B_{i,j}\right)$$

where set $B_{i,j}$ is a spherical surface depending on the bounds, i.e., $B_{i,j}=\{b|∥b∥=\frac{1}{2}\left(l_{i,j}+u_{i,j}\right)\}$.

A feasible approach is to find the convex envelope by relaxing constraint $∥b∥=d_{0}$ to $∥b∥\leq d_{0}$. Therefore, we obtained the convex form based on Function (8).

(11) $$\tilde{f}\left(a,d\right)=\underset{∥b∥\leq d_{0}}{\inf}{∥a-b∥}^{2}={\mathrm{dist}}^{2}\left(a,C\right)$$

where C={b|∥b∥≤d} is the convex hull of the non-convex set B.

According to Equation (11), we could establish the convex model corresponding to Problem (10):

(12) $$\min\;\sum_{\left(i,j\right)\in Z_{S}\cup Z_{A}}\frac{1}{2}{\mathrm{dist}}^{2}\left(x_{i}-x_{j},C_{i,j}\right)$$

where $C_{i,j}=\{b|∥b∥\leq \frac{1}{2}\left(l_{i,j}+u_{i,j}\right)\}$ is a convex set.

## 3. Distributed Framework

In order to approach the solution of convex optimization Problem (12) in a distributed way, we built the consensus form to formulate Problem (12) and design a parallel distributed algorithm based on ADMM. Problem (12) cannot be directly applied to parallel ADMM because it involves matrix calculation. We built the consensus form to decompose large-scale Problem (12) into N+M subproblems with independent variables. Then, we could design the ADMM framework to solve these subproblems in a parallel way. In this section, we prove the feasibility of decomposing Problem (12) and provide the concrete procedure of the parallel distributed algorithm.

The consensus form could independently represent the connections between each sensor and its neighbors in the whole network by duplicating a new vector of each node *j* at neighbor node *i*.

Let $z_{i}={\left[{\left(z_{i}\right)}_{j}^{T}\right]}^{T}\in R^{({\hat{N}}_{i}+1)d},i\in S\cup A$ be the local variable, where ${\hat{N}}_{i}$ represents the length of $N_{i}$. Component ${\left(z_{i}\right)}_{j}$ has the same dimension as $x_{i}$. Each of these local variables consists of selected elements of global variable $x={\left[x_{1}^{T},x_{2}^{T},\ldots ,x_{N+M}^{T}\right]}^{T}$. Mapping from the local variable to the global variable is

(13) $$\left\{ \begin{array}{c} {\left(z_{i}\right)}_{1}=x_{i}\\ {\left(z_{i}\right)}_{j}=x_{l} \end{array}i\in S\cup A,\;l\in N_{i},\;j=2,\ldots ,{\hat{N}}_{i}+1\right.$$

when we put the elements of neighbor set $N_{i}$ in corresponding vector $N_{i}$ in an ascending order, the relation between ${\left(z_{i}\right)}_{j}$ and $x_{l}$ is $l=N_{i}\left(j-1\right)$. $N_{i}\left(j\right)$ means the *j*th element of $N_{i}$. In other words, ${\left(z_{i}\right)}_{j}$ is the duplication of (j−1)th neighbor node at node *i*.

Therefore, Problem (12) has an equivalent form:

(14) $$\begin{array}{c} \min\;\sum_{i=1}^{N+M}\;\sum_{j=2}^{{\hat{N}}_{i}+1}\frac{1}{2}{\mathrm{dist}}^{2}\left({\left(z_{i}\right)}_{1}-{\left(z_{i}\right)}_{j},C_{i,l}\right)\\ \;s.t.\;z_{i}-{\tilde{x}}_{i}=0,\forall i\in S\cup A\\ \;\;{\left(z_{k}\right)}_{1}=x_{k},\forall k\in A \end{array}$$

where ${\tilde{x}}_{i}$ is the linear function of x.

We denoted local objective function as $F_{i}\left(z_{i}\right)=\sum_{j=2}^{{\hat{N}}_{i}+1}\frac{1}{2}{\mathrm{dist}}^{2}\left({\left(z_{i}\right)}_{1}-{\left(z_{i}\right)}_{j},C_{i,l}\right)$. Obviously, total objective function $F\left(z\right)=\sum_{i=1}^{N+M}F_{i}\left(z_{i}\right)$ is separable. Then, we could rewrite Problem (14) in another, simpler form:

(15) $$\begin{array}{c} \;\min\;\sum_{i=1}^{N+M}F_{i}\left(z_{i}\right)\\ s.t.\;z_{i}-A_{i}x=0,\forall i\in S\cup A\\ \;\;z\in R_{z} \end{array}$$

where $R_{z}$ is a linear space satisfying

(16) $$R_{z}=\left\{z|{\left(z_{k}\right)}_{1}=x_{k},\forall k\in A\right\}$$

Matrix $A_{i}$ indicates the linear relation between local variable $z_{i}$ and global variable x, of which the form is

(17) $$A_{i}=\left[\begin{array}{c} e_{1}\\ {\left(e_{l}\right)}_{\forall l\in N_{i}} \end{array}\right]⊗I_{d}$$

where $e_{l}$ is the *l*th unit-row vector in $R^{N+M}$, $I_{d}$ is an identity matrix of order *d*, and ⊗ is a Kronecker product.

By introducing an indicator function $G_{z}\left(z\right)$ of $R_{z}$, Problem (14) can be rewritten in a general consensus form as

(18) $$\begin{array}{c} \;\min\;F\left(z\right)+G_{z}\left(z\right)\\ s.t.\;z-Ax=0 \end{array}$$

where z is the shorthand notation for ${\left[z_{1}^{T},z_{2}^{T},\ldots ,z_{N+M}^{T}\right]}^{T}$, and $A=\left[A_{1};A_{2};\ldots ;A_{N+M}\right]$.

Before deriving the ADMM framework, we needed to build the augmented Lagrangian function of Problem (18)

(19) $$\begin{array}{cc} L(z,x,\lambda ;c) & =\left[F\left(z\right)+G_{z}\left(z\right)\right]+\lambda^{T}\left(z-Ax\right)+\sum_{i}\frac{1}{2}c{∥z_{i}-A_{i}x∥}^{2}\\ & =\sum_{i}\left[F_{i}\left(z_{i}\right)+G_{z_{i}}\left(z_{i}\right)+\lambda_{i}^{T}\left(z_{i}-A_{i}x\right)+\frac{1}{2}c{∥z_{i}-A_{i}x∥}^{2}\right] \end{array}$$

where λ is a vector consisting of Lagrangian multipliers, and *c* is the given penalty parameter. Indicator function $G_{z}\left(z\right)$ can be written as the sum of numerous subfunctions, namely, $G_{z}\left(z\right)=\sum_{i}G_{z_{i}}\left(z_{i}\right)$, by decomposing linear space $R_{z}$ as the Cartesian product of (N+M) closed convex sets $R_{z_{1}},R_{z_{2}},\ldots ,R_{z_{N+M}}$:

$$R_{z}=R_{z_{1}}\times R_{z_{2}}\times \ldots R_{z_{N}}\times R_{z_{N+1}}\times \ldots \times R_{z_{N}+M}$$

Indicator functions of sets $R_{z_{1}},\ldots ,R_{z_{N}}$ equal to identity matrices. Convex sets $R_{z_{N+1}},\ldots ,R_{z_{N+M}}$ have the affine form of

(20) $$R_{z_{k}}=\left\{z_{k}|T_{k}z_{k}=x_{k}\right\},\forall k\in A$$

where $T_{k}$ is a matrix satisfying $T_{k}={\left(1,\underset{N_{k}}{\underset{︸}{0,\ldots ,0}}\right)}^{T}⊗I_{d},k\in A$.

Thus, we can conclude that the objective function, constraints, and penalty term are separable for each sensor. Distributed optimization could be obtained by applying the ADMM method to Problem (18). The local updates are handled in an alternate way that can be separately optimized for variables z,x, and λ, as follows, for ∀i∈S∪A

(21) $$\begin{array}{c} z_{i}^{t+1}\in \underset{z_{i}}{\mathrm{argmin}}\left[F_{i}\left(z_{i}\right)+G_{z_{i}}\left(z_{i}\right)+\frac{1}{2}c{∥z_{i}-A_{i}x^{t}+{\tilde{\lambda }}_{i}^{t}∥}^{2}\right]\\ x^{t+1}\in \underset{x}{\mathrm{argmin}}\left(\sum_{i}\frac{1}{2}c{∥z_{i}^{t+1}-A_{i}x+{\tilde{\lambda }}_{i}^{t}∥}^{2}\right)\\ {\tilde{\lambda }}_{i}^{t+1}\in {\tilde{\lambda }}_{i}^{t}+c\left(z_{i}^{t+1}-A_{i}x^{t+1}\right) \end{array}$$

where $\tilde{\lambda_{i}}=\lambda_{i}/c$ is the scaled dual variable.

$z_{i}-$updates and ${\tilde{\lambda }}_{i}-$updates can be independently carried out in parallel for each node *i*. The concrete implementation steps are shown as follows.

(1) Initialize variables $z_{i}^{0}$, $x^{0}$, ${\tilde{\lambda }}_{i}^{0}$ for all nodes.

(2) Each node updates its local variables $z_{i}^{t+1}$ according to the first step of Problem (21), corresponding to the action

(22) $$z_{i}^{t+1}\in \underset{z_{i}}{\mathrm{argmin}}\left[F_{i}\left(z_{i}\right)+G_{z_{i}}\left(z_{i}\right)+\frac{1}{2}c{∥z_{i}-A_{i}x^{t}+{\tilde{\lambda }}_{i}^{t}∥}^{2}\right]$$

If we put indicate function $G_{z_{i}}\left(z_{i}\right)$ in the constraints, Problem (22) can be written in an equivalent form:

(23) $$\begin{array}{c} \;\min\left[\sum_{j=1}^{{\hat{N}}_{i}}\frac{1}{2}{\mathrm{dist}}^{2}\left({\left(z_{i}\right)}_{1}-{\left(z_{i}\right)}_{j},C_{i,j}\right)+\frac{1}{2}c\gamma_{i}\right]\\ s.t.\;∥z_{i}-A_{i}x^{t}+{\tilde{\lambda }}_{i}^{t}{∥}^{2}\leq \gamma_{i}\\ \;\;\gamma_{i}\geq 0\\ \;\;z_{i}\in R_{z_{i}} \end{array}$$

where $\gamma_{i}$ are the slack variables working as the quadratic penalty in the local cost function.

Obviously, Problem (23) is untypically convex. The gradient of $F_{i}\left(z_{i}\right)$ is discontinuous. Hence, classical methods such as the interior point method and the Newton method are not appropriate to solve these problems. In Section 4, we further derive an iterative algorithm to approach the solution of Problem (23). This iterative algorithm has the same convergence rate with the interior point method.

(3) Broadcast local variables $z_{i}^{t+1}$ to all nodes of neighbor sets.

(4) Each node computes variables ${\tilde{x}}_{i}^{t+1}$ according to

(24) $$x^{t+1}\in \underset{x}{\mathrm{argmin}}\left(\sum_{i}\frac{1}{2}c{∥z_{i}^{t+1}-A_{i}x+{\tilde{\lambda }}_{i}^{t}∥}^{2}\right)$$

Problem (24) is actually easy to solve by averaging all components of $z_{i}^{k+1}$ of which the local indices correspond to global index *l*. The x-update step has a simpler solution:

(25) $$x_{l}^{t+1}=\frac{1}{k_{l}}\left({\left(z_{l}\right)}_{1}^{t+1}+\sum_{\left(i,j\right)\in Z_{x_{l}}}{\left(z_{i}\right)}_{j}^{t+1}\right)$$

where $k_{l}$ is the number of local variable components corresponding to global components $z_{l}$ and $Z_{x_{l}}=\left\{\left(i,j\right)|N_{i}\left(j-1\right)=l\right\}$.

(5) Each node updates dual variables ${\tilde{\lambda }}_{i}^{t+1}$ according to

(26) $${\tilde{\lambda }}_{i}^{t+1}\in {\tilde{\lambda }}_{i}^{t}+c\left(z_{i}^{t+1}-A_{i}x^{t+1}\right)$$

**Figure 2:**
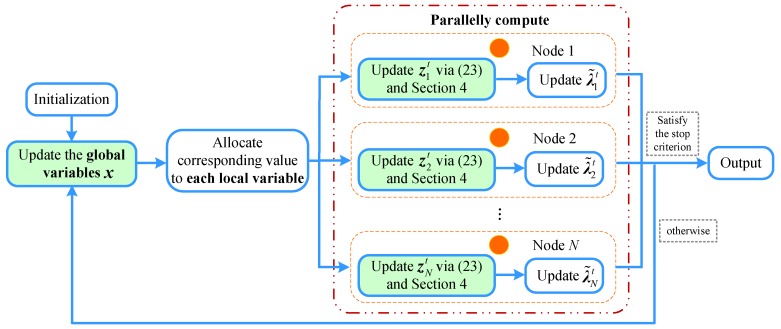
Flow chart of the ADMM_P algorithm.

A summary of the proposed parallel algorithm is illustrated as Algorithm 1 and Figure 2:

**Algorithm 1** ADMM_Projection (ADMM_P) algorithm for Problem (18). **Input**: number of anchors *M*, location of anchors $x_{k}\in R^{2},k\in A$, number of source sensors *N*, range measurements $\left\{r_{i,j},\left(i,j\right)\in Z_{\mathrm{NLOS}}\cup Z_{\mathrm{LOS}}\right\}$; **Output**: ${\left(z_{i}\right)}_{1},i\in S$
**Initialization**: t=0;
(1)Initialize local location of sensors $x^{0}$ and set dual variables $\lambda_{i}^{0}$ to zero;(2)According to $x_{i}^{0}$ and $\lambda_{i}^{0}$, compute the initial values:
$$z_{i}^{0}=A_{i}x^{0}$$**Updating iteration:**t++(1)Update local location variables $z_{i}^{t}$ at each node: ∀i∈A∪S
$$z_{i}^{t+1}\in \underset{z_{i}}{\mathrm{argmin}}\left[F_{i}\left(z_{i}\right)+G_{z_{i}}\left(z_{i}\right)+\frac{1}{2}c{∥z_{i}-A_{i}x^{t}+{\tilde{\lambda }}_{i}^{t}∥}^{2}\right]$$(2)Broadcast local variable $z_{i}^{t+1}$ to its neighbors $N_{i}$;(3)Update variable x according to Problem (25);(4)Update the local dual variable ${\tilde{\lambda }}_{i}^{t+1}$ according to Problem (26).


## 4. Solution to the Subproblem

Section 3 implies that the ADMM_P algorithm is an iterative method. Reference [27] guarantees that our algorithm converges to the same minimum as that in the centralized framework because its original counterpart (12) is convex. The algorithm summary indicates that the total computational complexity of ADMM_P almost relies on the local z-update (22). To satisfy the requirements on processing speed, we needed to design an effective algorithm that had both a fast convergence rate and low complexity. The Newton method and interior point method have these properties, but they were designed for smooth optimization problems [43]. Unfortunately, Problem (22) is a nonquadratic differential. Hence, we designed an iterative method based on the accelerated proximal algorithm, which is an analogous tool for nonsmooth optimization problems [21,44]. Given the structure of WSNs, we derived the proximal algorithm for source nodes and anchors, respectively.

### 4.1. Lipschitz Constant

The proximal algorithm needs the guarantee of Lipschitz continuity. First, we prove the premise in this subsection. To simplify notations, we defined the functions:

(27) $$\begin{array}{c} h\left(z_{i}\right)=\frac{1}{2}{\mathrm{dist}}^{2}\left(H_{i}z_{i},C_{i}\right)=\sum_{j=2}^{{\hat{N}}_{i}+1}\frac{1}{2}{\mathrm{dist}}^{2}\left({\left(z_{i}\right)}_{1}-{\left(z_{i}\right)}_{j},C_{i,l}\right)\\ g\left(z_{i}\right)=\frac{1}{2}c{∥z_{i}-A_{i}x^{t}+{\tilde{\lambda }}_{i}^{t}∥}^{2} \end{array}$$

where $C_{i}$ is Cartesian product of balls $C_{i,l},\;l\in N_{i}$, and $H_{i}$ has the form of

(28) $$\left[1_{N_{i}},-I_{N_{i}}\right]⊗I_{d}$$

Function $g(z_{i})$ in Functions (27) is convex and differentiable, and its gradient is

(29) $$\nabla g\left(z_{i}\right)=c\left[z_{i}-\left(A_{i}x^{t}-{\tilde{\lambda }}_{i}^{t}\right)\right]$$

Obviously, this gradient is Lipschitz-continuous, and we could obtain its Lipschitz constant as follows

(30) $$\begin{array}{c} \begin{array}{cc} ∥\nabla g(x)-\nabla g(y)∥ & =∥cx-cy∥\\ & \leq k_{g}|c|∥x-y∥ \end{array}\\ \Rightarrow \frac{∥\nabla g(x)-\nabla g(y)∥}{∥x-y∥}\leq k_{g}\left|c\right|=L_{g},\forall x,y\in R^{d({\hat{N}}_{i}+1)} \end{array}$$

where $k_{g}\geq 1$ is a constant.

### 4.2. Source Nodes

For source nodes, local Subproblem (22) has the equivalent simpler form

(31) $$\min h\left(z_{i}\right)+g\left(z_{i}\right)$$

the proximal mapping of function $h\left(z_{i}\right)=\frac{1}{2}{\mathrm{dist}}^{2}\left(H_{i}z_{i},C_{i}\right)$ is

(32) $${\mathrm{prox}}_{\mu h}\left(v_{i}\right)=H_{i}^{†}\left[H_{i}v_{i}+\frac{\mu }{1+\mu }\left(P_{C_{i}}\left(H_{i}v_{i}\right)-H_{i}v_{i}\right)\right]$$

where $\mu =1/L_{g}$ is the step size, $H_{i}^{†}$ is the pseudoinverse of $H_{i}$, and $P_{C_{i}}\left(H_{i}z_{i}\right)$ is the orthogonal projection of point $H_{i}z_{i}$ onto the convex set $C_{i}$, i.e.,

(33) $$P_{C_{i}}\left(H_{i}z_{i}\right)\in \arg\min\{∥x-H_{i}z_{i}∥\;|x\in C_{i}\}$$

Hence, we can obtain the accelerated proximal algorithm for local Subproblems (31), as follows, for k≥1:

(34) $$\begin{array}{c} \begin{array}{cc} \omega_{i}^{\left(k-1\right)} & =\xi_{i}^{\left(k-1\right)}-\mu_{k}\nabla g\left(\xi_{i}^{\left(k-1\right)}\right)\\ & =\xi_{i}^{\left(k-1\right)}-\mu_{k}c\left[\xi_{i}^{\left(k-1\right)}-\left(A_{i}z^{t}-{\tilde{\lambda }}_{i}^{t}\right)\right] \end{array}\\ \eta_{i}^{\left(k\right)}=\frac{1}{1+\mu_{k}}\omega_{i}^{\left(k-1\right)}+\frac{\mu_{k}}{1+\mu_{k}}H_{i}^{†}P_{C_{i}}\left(H_{i}\omega_{i}^{\left(k-1\right)}\right)\\ \xi_{i}^{\left(k\right)}=\eta_{i}^{\left(k\right)}+\frac{k-1}{k+2}\left(\eta_{i}^{\left(k\right)}-\eta_{i}^{\left(k-1\right)}\right) \end{array}$$

This iterative procedure stops when the stopping criterion is satisfied. $\mu_{k}$ is the step size. Here, we used the fixed step size $\mu_{k}=\mu =1/L_{g}$. We used the estimation results of the last loop as the initial point of this subupdate, i.e., $\xi_{i}^{\left(0\right)}=z_{i}^{t}$. The subupdate outputs $\xi_{i}^{\left(k\right)}=z_{i}^{t+1}$ as the local estimation result.

### 4.3. Anchor Nodes

For anchor nodes ${\left(z_{i}\right)}_{1}=x_{i},i\in A$, Problem (22) becomes an equality-constrained optimization problem for i≥N+1,

(35) $$\begin{array}{c} \;\min h\left(z_{i}\right)+g\left(z_{i}\right)\\ s.t.\;\;T_{i}z_{i}=x_{i} \end{array}$$

Our approach solving this problem was to eliminate the equality constraint via the equation

(36) $$\left\{z_{i}|T_{i}z_{i}=x_{i}\right\}=\left\{Q_{i}\beta_{i}+\hat{z_{i}}|\beta_{i}\in R^{N_{i}d}\right\}$$

where $\hat{z_{i}}={\left(x_{i}^{T},\underset{N_{i}d}{\underset{︸}{0,\ldots ,0}}\right)}^{T}$, $Q_{i}$ has the form of

(37) $$Q_{i}=\left[\begin{array}{cc} e_{1}^{d}-e_{2}^{d} & 0_{1}\\ 0_{2} & I_{N_{i}d} \end{array}\right]$$

where $e_{i}^{d},i=1\;or\;2$ is the *i*th unit vector in $R^{d}$, $I_{N_{i}d}$ is an identity matrix with order $N_{i}d$, and $0_{i},i=1\;or\;2$ is a zero matrix.

With Equation (36), we form eliminated optimization problem

(38) $$\begin{array}{cc} & \min\;\hat{h}\left(\beta_{i}\right)+\hat{g}\left(\beta_{i}\right)\\ \Rightarrow & \min\;h\left(Q_{i}\beta_{i}+\hat{z_{i}}\right)+g\left(Q_{i}\beta_{i}+\hat{z_{i}}\right) \end{array}$$

which is an unconstrained problem with variable $\beta_{i}$. Then, we could derive the solution of equality-constrained Problem (35) according to Equations (34).

Introducing the affine structure does not change the convexity and continuity of the functions. The gradient of the $\hat{g}\left(\beta_{i}\right)$ function is still Lipschitz-continuous. The gradient of $\hat{g}\left(\beta_{i}\right)$ is

(39) $$\nabla \hat{g}\left(\beta_{i}\right)=c\left[Q_{i}^{T}Q_{i}\beta_{i}+Q_{i}^{T}\left({\hat{z}}_{i}-A_{i}x^{t}+{\tilde{\lambda }}_{i}^{t}\right)\right]$$

Hence, the new Lipschitz constant could be obtained by the following inequality:

(40) $$\begin{array}{c} \begin{array}{cc} ∥\nabla \hat{g}\left(x\right)-\nabla \hat{g}\left(y\right)∥ & =∥cQ_{i}^{T}Q_{i}x-cQ_{i}^{T}Q_{i}y∥\\ & =|c|∥Q_{i}^{T}Q_{i}\left(x-y\right)∥\\ & \leq \left|c\right|\nu_{\max}\left(Q_{i}^{T}Q_{i}\right)∥x-y∥ \end{array}\\ \Rightarrow \frac{∥\nabla \hat{g}\left(x\right)-\nabla \hat{g}\left(y\right)∥}{∥x-y∥}\leq \left|c\right|\nu_{\max}\left(Q_{i}^{T}Q_{i}\right)=L_{\hat{g}},\forall x,y\in R^{d{\hat{N}}_{i}} \end{array}$$

where $\nu_{\max}\left(Q_{i}^{T}Q_{i}\right)$ is the maximal eigenvalue of $Q_{i}^{T}Q_{i}$.

By using the conclusion of Equation (32) to Equation (34),the accelerated proximal algorithm for the local subproblem of the anchor nodes is presented as follows:

(41) $$\begin{array}{c} \omega_{i}^{\left(k-1\right)}=\xi_{i}^{\left(k-1\right)}\;-\mu_{k}c\left[Q_{i}^{T}Q_{i}\xi_{i}^{\left(k-1\right)}+Q_{i}^{T}\left({\hat{z}}_{i}-A_{i}x^{t}+{\tilde{\lambda }}_{i}^{t}\right)\right]\\ \eta_{i}^{\left(k\right)}=\frac{{\hat{\mu }}_{k}}{1+{\hat{\mu }}_{k}}\left(\omega_{i}^{\left(k-1\right)}+Q_{i}^{†}\hat{z_{i}}\right)+\frac{{\hat{\mu }}_{k}}{1+{\hat{\mu }}_{k}}{\left(H_{i}Q_{i}\right)}^{†}P_{C_{i}}\left(H_{i}\left(Q_{i}\omega_{i}^{\left(k-1\right)}+\hat{z_{i}}\right)\right)\\ \xi_{i}^{\left(k\right)}=\eta_{i}^{\left(k\right)}+\frac{k-1}{k+2}\left(\eta_{i}^{\left(k\right)}-\eta_{i}^{\left(k-1\right)}\right) \end{array}$$

## 5. Numerical Simulations

In this section, we present the simulation results to demonstrate the strengths and weaknesses of the ADMM_P algorithm. We considered the worst case, where NLOS connections are unidentifiable and the probability of these NLOS connections is up to 95%. We preliminarily considered a network that had 10 anchor nodes and 40 source nodes randomly distributed in a [−50 m, 50 m] × [−50 m, 50 m] square. Noisy range measurements were available when distances $r_{i,j}\leq 30$ m. The coordinate dimension is d=2. Range measurements were generated according to Equations (1) and (2),where NLOS error $\varepsilon_{i,j}$ is exponentially distributed with mean parameter $\alpha_{\mathrm{NLOS}}$. For a noisy environment, we assumed that the measurement noise was independent and identically distributed, i.e., $\sigma_{i,j}=\sigma_{n}$.

We compared the accuracy of ADMM_P with four state-of-art algorithms, which are named:

(1) SDP: SDP method of Reference [36] designed for NLOS environments;
(2) SDPH: SDP model based on a heuristic solution [35];
(3) EDM: EDM model based on three-block ADMM [37];
(4) ADMM_SF: the parallel algorithm of Reference [20], which was only designed for LOS environments.

It is worth noting that ADMM_P is an iterative algorithm working in the parallel distributed framework, and that there are few authoritative publications about distributed algorithms for NLOS localization. Therefore, we only analyzed the convergence property for our algorithm. In the convergence analysis, we provide the estimation results of SDPH of Reference [35] as references.

All simulations were carried out in MATLAB. Local convex Problems (22) were handled via the MATLAB Optimization Toolbox with fmincon solver. Algorithm performance was evaluated via Cumulative Distribution Function (CDF), Root Mean Square Error (RMSE), and objective Function Value (FVAL):

(42) $$\begin{array}{c} \mathrm{RMSE}=\sqrt{\frac{\sum_{i\in S}{∥{\left(z_{i}\right)}_{1}-p_{i}∥}^{2}}{N}}\\ \mathrm{FVAL}=\sum_{i\in S}\left[F_{i}\left(z_{i}\right)+\frac{1}{2}c_{i}{∥z_{i}-A_{i}x+{\tilde{\lambda }}_{i}∥}^{2}\right] \end{array}$$

where $p_{i}$ is the true position, and ${\left(z_{i}\right)}_{1}$ is the estimated location of node *i*.

### 5.1. Performance Comparison

In this part, we verify the superiority of our method in the aspects of localization accuracy and computational complexity, respectively. We fixed the number of source nodes and anchors as *N* = 40, *M* = 10. The mean parameter of NLOS propagation is $\alpha_{\mathrm{NLOS}}=3$. Sensor nodes (including anchors) were randomly distributed in the square of [−50m,50m]×[−50m,50m]. To show the simulation results more precisely, we compared the ADMM_P algorithm with three other state-of-art methods in the same simulation environment.

#### 5.1.1. Accuracy Comparison

**Figure 3:**
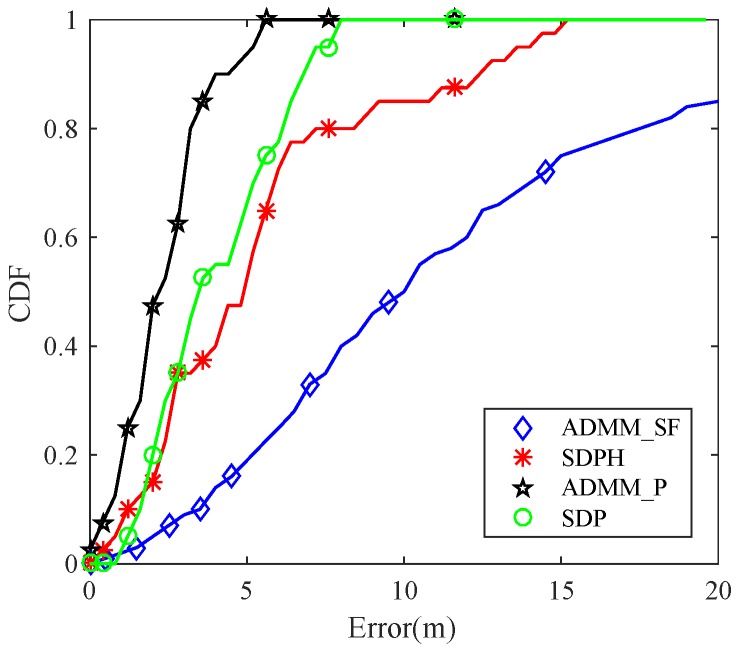
Cumulative Distribution Function (CDF) comparison of the four aforementioned methods for a M=10,N=40 sensor network. Anchors were randomly distributed. Probability of NLOS connections was set to $P_{NLOS}=95\%$.

**Figure 4:**
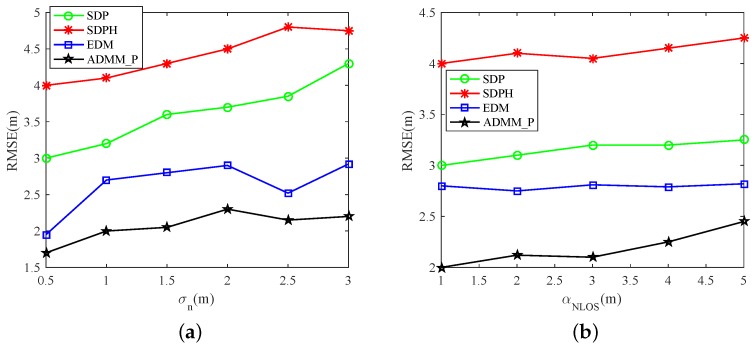
Performance comparison in varying environments with a M=10,N=40 sensor network. Probability of NLOS connections was set to $P_{NLOS}=95\%$. (**a**) Root Mean Square Error (RMSE) comparison in varying noise environments; (**b**) RMSE comparison with different NLOS parameters.

Figure 3 and Figure 4 show the simulation results of the four available methods. As is shown, ADMM_P had higher accuracy than other three methods. In Figure 3, the CDF curve of ADMM_P is in the leftmost side of the three other methods. This means that the estimation results of ADMM_P were more concentrated around lower errors. Especially at point Error=2.5m, the CDF of our method improved by up to 50% compared with the ADMM_SF, which did not utilize any NLOS mitigation techniques. In Figure 4, we further analyzed the performance of the four methods in varying environments with different $\sigma_{n}$ and $\alpha_{\mathrm{NLOS}}$, respectively. The simulation results show that ADMM_P has higher accuracy, and performance is more stable in different environments.

#### 5.1.2. Complexity Analysis

We compared computational complexity in Table 1, in which we used $N_{c}$ to represent the connective numbers of pair nodes $\left(i,j\right)\in Z_{S}\cup Z_{A}$. We could find that ADMM_P is more suitable for practical situations because it has much lower computational complexity and smaller problem size. The parallel distributed framework plays a key role in decreasing complexity.

The SDPH method in Reference [35] and the SDP method in Reference [36] were implemented in the centralized framework. The problem sizes of SDPH and SDP were, respectively, dN+1/2∗N(N+1) and $dN+1/2N\left(N+1\right)+N^{2}$, both depending on the whole scale of the WSN *N*. The EDM method in Reference [37] applied a three-block ADMM, but problem size also depends on *N*. According to Table 1, we can see that the problem size of the ADMM_P algorithm was determined by the decomposed local minimization and could be reduced to $d\left({\hat{N}}_{i}+1\right)+{\hat{N}}_{i}$, where ${\hat{N}}_{i}$ represents the number of neighbors. Because of the limitation of $r_{\mu }$, the size of the neighbor set was much smaller than the whole scale of the WSN, i.e., ${\hat{N}}_{i}\ll N$. This conclusion can theoretically prove that our method can drastically reduce the precessing burden, no matter the problem size or computational complexity.

Our method decomposes the original large-scale problem into N+M small-scale subproblems via consensus form, and handles these subproblems in a parallel way. Hence, when compared with the centralized algorithm, our method can dramatically decrease computational complexity. In Section 3 and Section 4, we could find that the total complexity of the proposed algorithm almost depends on solving the subproblems. Section 4 provides an iterative method to solve the subproblems, which are nonquadratic differentials. In this iterative method, with the guarantee of objective-function convexity, the optimal value is finite and attained at $z_{i}^{*}$. The derived method is known to converge at a rate of $O(1/k^{2})$ [44]:

(43) $$F_{i}\left(z_{i}^{\left(k\right)}\right)-F_{i}^{*}\leq \frac{2}{{\left(k+1\right)}^{2}\mu }{∥z_{i}^{\left(0\right)}-z_{i}^{*}∥}^{2}$$

Subupdates require very simple operations, no matter if they are at anchor nodes or at source nodes. The inversion of matrix $H_{i}$ and $H_{i}Q_{i}$ is easy to precompute because $H_{i}$ and $Q_{i}$ only consist of identity matrices and unit vectors. Projection on set $C_{i}$ can be decomposed into projections on sets $C_{i,j},j\in N_{i}$, which involves very limited dimension d≤3 and is easy to compute. Therefore, the local update given by Equations (34) and (41) has much lower complexity than the three other algorithms. However, because every node shares the processing burden and plays the same role in the parallel framework, the communication cost of each node is higher than that of centralized frameworks.

### 5.2. ADMM_P Properties in Different Noisy Environments

**Figure 5:**
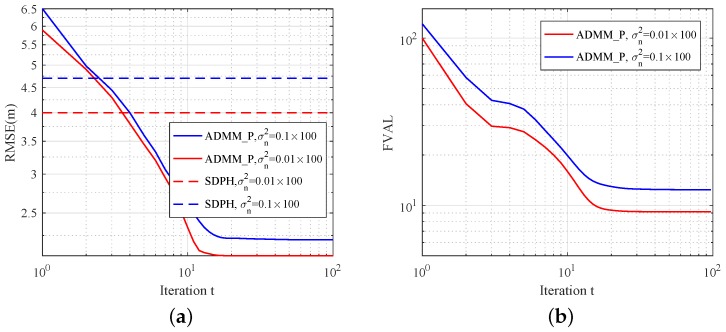
Estimation results with different $\sigma_{n}^{2}$ for N=40,M=10 sensor network versus iteration number *t*. Probability of NLOS connections was set to $P_{NLOS}=95\%$. (**a**) RMSE; (**b**) convergence properties.

In this scenario, we fixed the number of anchors and the distribution parameter of NLOS errors to show localization accuracy and convergence property in different noisy environments. The mean parameter of NLOS error was fixed as $\alpha_{\mathrm{NLOS}}=3$. Ten anchors were randomly deployed in the region of 100 × 100 m. As is shown in Figure 5a,b, we present the simulation results with $\sigma_{n}^{2}=0.01\;\times \;100$ and $\sigma_{n}^{2}=0.1\times 100$. We found that ADMM_P mitigates the effect of NLOS range measurements and provides better estimation accuracy. SDPH is solved with the interior-point method under a centralized framework, so its simulation results are only provided as accuracy references in Figure 5. From the figure, we can see that ADMM_P has good convergence performance in different noisy environments and higher accuracy than SDPH. In Figure 5b, we further analyzed the convergence performance of ADMM_P. Given that the local objective functions are based on heuristic solutions, the function value cannot converge to an extremely low level. Hence, the curve tendencies in Figure 5b still demonstrate a stable convergence of ADMM_P.

### 5.3. ADMM_P Properties in Different NLOS Environments

**Figure 6:**
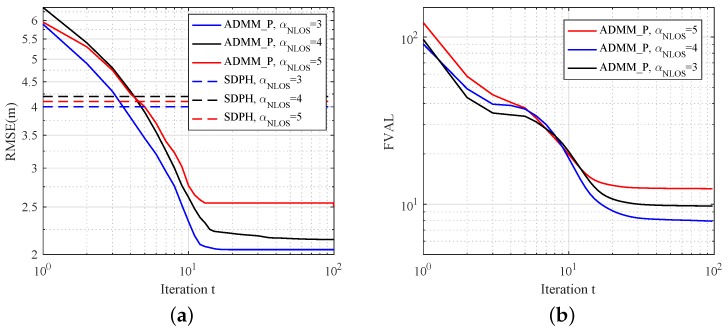
Estimation results with different $\alpha_{\mathrm{NLOS}}$ for N=40,M=10 sensor network versus iteration number *t*. Probability of NLOS connections was set to $P_{NLOS}=95\%$. (**a**) RMSE; (**b**) convergence properties.

In this scenario, we fix the number of anchors and the noise level to study ADMM_P performance with different NLOS error distributions. The measurement noise variance was fixed as $\sigma_{n}^{2}=0.01\times 100$. Other simulation settings were the same as those in Scenario B. In Figure 6a,b, we compared the performance of ADMM_P with $\alpha_{\mathrm{NLOS}}=3,4,5$. As we can see, although RMSE slightly increased as mean parameter $\alpha_{\mathrm{NLOS}}$ grew, the accuracy of ADMM_P is still better than SDPH. This also indicates that the proposed algorithm effectively mitigates NLOS error. Simulation results in Figure 6b verify the stable convergence performance of ADMM_P as varying NLOS error distribution.

### 5.4. ADMM_P Algorithm Properties with Different Anchor Placements

#### 5.4.1. Anchor Placement

**Figure 7:**
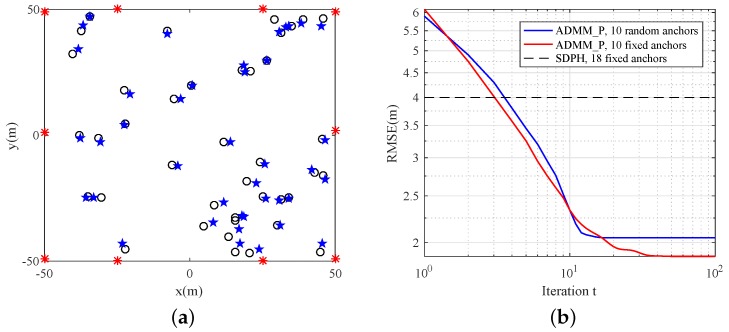
Estimation results of a sensor network with 10 fixed anchors and 40 randomly distributed sensors. (**a**) Estimation locations. Red asterisks *: anchor locations; black circles ⚬: true sensor locations; blue stars ★: estimation results; (**b**) RMSE comparison.

In this scenario, we deployed the anchors at the perimeter of the region. We set $\sigma_{n}^{2}=0.01\times 100$ and $\alpha_{\mathrm{NLOS}}=3$. We compared localization accuracy when the anchor placement was random or fixed. In Reference [13], the author proposed that an appropriate anchor-placement deign could further improve localization accuracy. Hence, we placed the anchors around the perimeter of the region to avoid getting crowded points, so that the higher dimensional projection of anchors could contain more sensor nodes. The corresponding simulation results are shown in Figure 7b, from which we can see that the fixed anchor placement improves the localization performance of the ADMM_P algorithm in the same NLOS environment. Furthermore, in the scenario of fixed anchors, the accuracy of the ADMM_P algorithm is higher than that of the SDPH algorithm, even if the former employs the less anchors.

#### 5.4.2. Varying Numbers of Anchors

**Figure 8:**
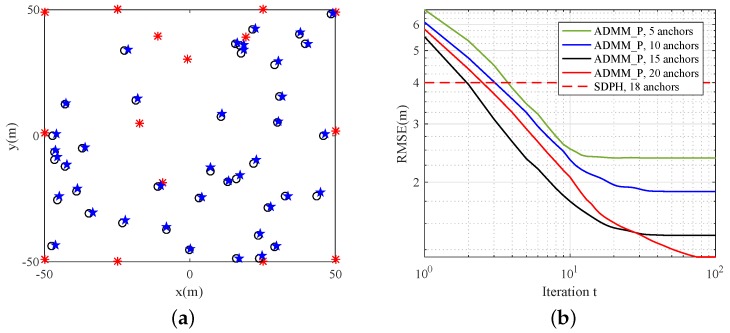
Estimation results of sensor network with 10 fixed anchors, five randomly distributed anchors, and 40 randomly distributed sensors. (**a**) Estimation locations. Red asterisks *: anchor locations; black circles ⚬: true sensor locations; blue stars ★: estimation results; (**b**) RMSE comparison.

Simulations in the last subsection show that a predesigned placement of anchors has a positive influence on localization performance. In this scenario, we evaluated the performance of the ADMM_P algorithm by varying the number of anchors. Taking the simulation results of Figure 7 into consideration, we fixed 10 anchors on the region boundary and randomly deployed the remaining anchors inside the region. Other simulation settings were the same as those in the last subsection. Node distribution is shown in Figure 8a, and the corresponding simulations are shown in Figure 8b, which shows that proper anchor placement and an increase in anchor number both lead to an improvement of localization performance.

## 6. Conclusions

In this paper, we proposed an efficient distributed algorithm for NLOS cooperative localization. We employed tight convex relaxation for the heuristic model, and approached it in a parallel ADMM framework to offer powerful computation ability. The distributed framework can drastically reduce the computational burden since it decomposes a large-scale problem into numerous small-scale problems, which similarly retains computational time when the number of sensors grows. Furthermore, we derived the accelerated proximal gradient method to improve the convergence rate of local subproblems. Simulation results demonstrate that our method efficiently alleviates the influence of NLOS propagation and has fairly good convergence performance.

In future works, we plan to study the level of influence that step size and penalty parameters could have on the performance of local subproblems and an ADMM framework. How to establish the mathematic model to find the optimal parameters needs further study as well. Moreover, the lower and upper bounds of range measurements provide a good feasible region, which is nevertheless nonconvex. We are interested in finding better convex relaxation rather than utilizing a heuristic solution as the optimal solution.

## Figures and Tables

**Table 1:** Complexity comparison of available algorithms.

	SDPH [35]	SDP [36]	EDM [37]	ADMM_P
Convex problem size	dN+1/2N(N+1)	$dN+1/2N\left(N+1\right)+N^{2}$	$N+M\left(M-1\right)/2+N_{c}$	$d({\hat{N}}_{i}+1)$
Computational complexity	$O(N^{3})$	$O({\left(2N\right)}^{3})$	$O(N^{3}+N^{2})$	$O(d^{3}+{\left(d{\hat{N}}_{i}\right)}^{2})$
Type of framework	Centralized SDP	Centralized SDP	Three-block ADMM	Parallel ADMM
Communication cost	*N*	*N*	*N*	$d({\hat{N}}_{i}+1)$
